# The clinical utility of next generation sequencing in endometrial cancer: focusing on molecular subtyping and lynch syndrome

**DOI:** 10.3389/fgene.2024.1440971

**Published:** 2024-09-05

**Authors:** Yongzhen Guo, Guangwei Yan, Pei Zhang, Yixuan Liu, Chengquan Zhao, Xianxu Zeng

**Affiliations:** ^1^ Department of Pathology, The Third Affiliated Hospital of Zhengzhou University, Zhengzhou, China; ^2^ Zhengzhou Key Laboratory of Gynecological Disease’s Early Diagnosis, Zhengzhou, China; ^3^ Department of Pathology, Magee-Womens Hospital, University of Pittsburgh Medical Center, Pittsburgh, PA, United States

**Keywords:** endometrial cancer, next generation sequencing, molecular subtyping, lynch syndrome, clinical pathological characteristics

## Abstract

**Objective:**

To investigate the clinical utility of Next Generation Sequencing (NGS) in molecular typing of endometrial carcinoma and its combined screening for Lynch Syndrome (LS).

**Methods:**

90 patients diagnosed with endometrial carcinoma (EC) and receiving treatment at the Third Affiliated Hospital of Zhengzhou University between March 2022 and December 2023 were included in this study. Molecular typing and germline evaluation for LS were conducted using NGS on the Illumina platform. A retrospective analysis was performed to examine the clinical pathological characteristics, molecular mutation spectrum, and LS screening outcomes among patients with four distinct molecular subtyping categories.

**Results:**

Among the 90 cases of EC, 11 cases (12.2%) of *POLE* mut type, 19 cases (21.1%) of MMRd type, 6 cases (6.7%) of p53 abn type, and 54 cases (60%) of NSMP type were detected, with detailed analysis of their respective molecular characteristics. LS screening identified 9 cases (10%) of pathogenic germline mutations in MMR genes, including 3 cases of *MLH1* germline mutations, 2 cases of *PMS2*, 2 of *MSH2*, and 2 of *MSH6*. Of the 9 LS patients, 7 were MMRd type and 2 NSMP type, with 7 cases showing abnormal MMR protein expression. Additionally, 6 cases with germline variants of uncertain significance in MMR genes were detected, including 2 *MLH1*, 1 *MSH6*, 2 *MSH6*, 1 *PMS2*, and 1 *EPCAM*.

**Conclusion:**

NGS enables precise molecular typing of endometrial carcinoma through the identification of mutations in the *POLE*, *TP53*, and MMR genes. Conducting germline mutation testing for MMR genes in all patients with endometrial carcinoma can effectively prevent instances of overlooked LS diagnoses. Nevertheless, the extensive expenses associated with NGS necessitate additional validation and investigation before its clinical implementation can be fully endorsed.

## Introduction

EC is one of the most common gynecological malignancies and ranks as the second most prevalent cancer in the female reproductive system in China (Gu et al., 2021). Traditional histological classification of EC has been augmented by molecular subtyping strategies proposed by The Cancer Genome Atlas (TCGA) in 2013. TCGA’s comprehensive multi-omics study categorized EC into four distinct molecular subtypes: *POLE* (ultramutated), MSI (hypermutated), copy number high (serous-like), and copy-number low (endometrioid), which are instrumental in assessing prognosis and recurrence risks (Levine et al., 2013). TCGA utilized genome technologies such as high-throughput sequencing and single nucleotide polymorphism microarrays, posing challenges in clinical implementation. Subsequent studies have continuously refined molecular subtyping methods for EC, with current prevalent techniques including ProMisE and Tans-PORTEC subtyping (Talhouk et al., 2015; Stelloo et al., 2015).

EC is the most common extracolonic tumor and a sentinel cancer in females with LS. Approximately half of the women with LS present EC as their initial malignancy. LS-associated EC (LS-EC) is caused by pathogenic germline mutations in mismatch repair (MMR) genes including *MLH1*, *MSH2*, *MSH6*, *PMS2*, and *EPCAM*. Laboratory screening methods for LS-EC encompass immunohistochemistry, *MLH1* methylation testing, MSI testing, with the definitive diagnosis of LS-EC requiring NGS to detect germline mutations in MMR genes (Zhao et al., 2022).

Currently, there is a gap in clinical practice regarding the genetic risk screening workflow, the choice of molecular subtyping strategies, and the understanding of LS-EC. This study engaged 90 EC patients from the Third Affiliated Hospital of Zhengzhou University. It utilized NGS for molecular subtyping and LS screening, aiming to explore the clinical utility of NGS in the combined molecular subtyping and LS screening in EC.

## Methods

### Clinical data collection

From March 2022 to December 2023, a total of 90 patients diagnosed with EC at the Third Affiliated Hospital of Zhengzhou University were included in this study. Each case underwent routine Hematoxylin and Eosin (H&E) and immunohistochemical (IHC) staining. The slides were independently reviewed and diagnosed by two qualified pathologists. Collected clinical and pathological data included age, body mass index (BMI), menopausal status, obstetric history, familial history of malignant neoplasm (MN-FH), histological type, FIGO stage (2009 edition), myometrial invasion depth, metabolic syndrome comorbidities (including hypertension, diabetes, coronary heart disease, hypothyroidism, etc.), lymph node metastasis, lymph vascular space invasion (LVSI), and relevant immunohistochemical markers (including *MLH1*, *MSH2*, *MSH6*, *PMS2*, *TP53*). The study was approved by the Ethical Committee of Zhengzhou University’s Third Affiliated Hospital (Approval No: 2022-362-01) and informed consent was obtained from all patients.

### IHC

Tumor tissue samples were fixed in 3.7% neutral formaldehyde, dehydrated, and embedded in paraffin. Sections were stained using H&E and immunohistochemistry. The EnVision two-step immunohistochemistry staining method was used, with diaminobenzidine (DAB) as the chromogen, following the procedures and reagent instructions of the Ventana Benchmark Ultra automatic immunohistochemistry instrument (Roche Ventana). Antibodies used included *MLH1*, *MSH2*, *MSH6*, *PMS2*, and *TP53*, all procured from Beijing Zhongshan Golden Bridge Biotechnology Co., Ltd.

### Molecular subtyping process

Tumor-rich tissue wax blocks were selected from H&E slides for microtome sectioning. Detection was carried out using NGS technology based on the Illumina platform. Briefly, DNA was extracted from formalin-fixed paraffin-embedded (FFPE) tissue samples and quantified using a nucleic acid quantification instrument. Library preparation included pre-denaturation, hybridization, extension and ligation, enzyme digestion, and library amplification. The library was then purified using magnetic beads. High-throughput sequencing was performed using an Illumina sequencing platform. Sequencing data analysis software was used to identify mutations in target genes and assess MSI. The scope of testing included the exonuclease domain of the *POLE* gene (Exon 3-14) and part of Exon 19, the entire coding region of the *TP53* gene, and 55 microsatellite loci (MS). Detected mutations included point mutations, small fragment insertions/deletions (InDels), and MSI in the targeted regions of *POLE* and *TP53*. Molecular subtyping results included: *POLE* mutation type (*POLE* mut), mismatch repair deficiency type (MMRd), non-specific molecular profile (NSMP), and p53 mutation type (p53 abn). Genetic variants were classified according to their clinical significance into four categories: Class I (clinically significant with Level A or B evidence), Class II (potentially clinically significant with Level C or D evidence), Class III (uncertain clinical significance), and Class IV (benign or likely benign) (Li et al., 2017). The specific molecular subtyping was determined based on the following principles: First, the *POLE* gene mutation status was assessed. If pathogenic mutations in the *POLE* gene were detected, the sample was classified as *POLE* mut. If the *POLE* gene was wild-type or had non-pathogenic mutations, the MSI status was then evaluated. If MSI-H was present, the sample was classified as MMRd. If the MSI status was MSS, the *TP53* status was further assessed. If *TP53* mutations were detected, the sample was classified as p53abn. If *TP53* was wild-type, the sample was classified as NSMP.

### LS screening

MMR gene germline mutation testing was conducted using NGS technology based on the Illumina platform with blood samples from patients. DNA is extracted from peripheral blood samples and quantified using a fluorescence-based nucleic acid quantification method, ensuring a concentration of at least 3.75 ng/µL and a total amount of no less than 30 ng. The DNA is then subjected to a pre-denaturation reaction, where it is heated to 98°C for 5 min and then immediately placed on ice for 1 min. Following this, the DNA is hybridized with specific probes in a hybridization buffer at 95°C for 5 min, 60°C for 2 h, and then held at 4°C. After hybridization, the extension and ligation reaction is performed at 60°C for 10 min, followed by a hold at 4°C. The DNA is then subjected to an exonuclease digestion reaction with Exonuclease A and B at 37°C for 30 min and 95°C for 10 min, followed by a hold at 4°C. The library is amplified using PCR with a pre-mixed PCR solution and specific primers under the following conditions: 98°C for 1 min, 98°C for 20 s, 61°C for 30 s, and 72°C for 20 s, repeated for 25 cycles, followed by 72°C for 5 min and a final hold at 4°C. The amplified library is purified using magnetic beads, washed twice with 80% ethanol, and eluted in Low TE solution. The quality of the library is assessed by measuring its concentration using a fluorescence-based method, ensuring it is at least 10 ng/µL, and by fragment analysis using a Bioanalyzer, ensuring the fragments are primarily between 260 and 360 bp. Finally, sequencing is performed on an Illumina platform using a 300-cycle paired-end read (2 × 150 cycles) kit, with a minimum sequencing output of 50 Mb and a 2% spike-in of PhiX Control v3. The range of detection included the coding regions and exon-intron junctions (±10 bp) of the *EPCAM*, *MLH1*, *MSH2*, *MSH6*, and *PMS2* genes. Detected mutation types included single nucleotide variations (SNVs) and small fragment insertions/deletions. The results were categorized into five types: benign, likely benign, variant of uncertain significance, likely pathogenic, and known pathogenic mutations. A diagnosis of LS-associated EC (LS-EC) was confirmed when results indicated likely pathogenic or pathogenic germline mutations.

## Results

### Analysis of clinical and pathological data of different molecular subtypes of EC

In this study, 90 patients with EC were included. The mean age was 53.3 years (±10.7). A family history of malignant tumors was present in 10 patients, and 57 patients had metabolic syndrome, with 30 of these having a history of hypertension. Molecular typing was performed using Next-Generation Sequencing (NGS) methods. The breakdown of the molecular subtypes was as follows: *POLE* mutation in 11 cases (12.2%), MMR deficiency (MMRd) in 19 cases (21.1%), p53 abnormality in 6 cases (6.7%), and No Specific Molecular Profile (NSMP) in 54 cases (60%). According to the 5th edition of the WHO Classification of Female Reproductive Organ Tumors, 83 cases were classified as endometrioid adenocarcinoma; 4 as serous carcinoma, of which 3 were NSMP and 1 was p53 abnormal; 1 case as clear cell carcinoma, and 1 as middle nephric adenocarcinoma, both NSMP; and 1 case of dedifferentiated carcinoma was MMRd. According to FIGO staging, 72 cases were in stages I-II, and 18 in stages III-IV. The p53 mutation subtype exhibited more severe muscular invasion, vascular invasion, and lymphatic metastasis compared to other types, as detailed in Table 1.

**TABLE 1:** Clinical and pathological characteristics between different molecular subtypes of EC.

Molecular classification		*POLE* mut	MMRd	*p53* abn	NSMP	All
N		11 (12.2%)	19 (21.1%)	6 (6.7%)	54 (60%)	90
Age (years)		57.5 (5.3)	53.7 (7.0)	57.50 (11.2)	51.9 (12.2)	53.3 (10.7)
		56 (51–70)	54 (36–66)	57 (40–70)	55 (24–71)	55 (24–71)
Height (cm)		160 (4.8)	160 (3.6)	165 (7.8)	160 (4.8)	160 (4.9)
Weight (kg)		66.32 (14.09)	64.08 (6.68)	68.25 (13.39)	63.56 (11.86)	64.32 (11.27)
MN-FH						
	Yes	1 (9.09%)	3 (15.79%)	1 (16.67%)	5 (9.26%)	10 (11.11%)
	No	10 (90.91%)	16 (84.21%)	5 (83.33%)	49 (90.74%)	80 (88.89%)
Gravidity						
	0	0 (0.00%)	0 (0.00%)	0 (0.00%)	9 (16.67%)	9 (10.00%)
	1	1 (9.09%)	1 (5.26%)	1 (16.67%)	10 (18.52%)	13 (14.44%)
	2+	10 (90.91%)	18 (94.74%)	5 (83.33%)	35 (64.81%)	68 (75.56%)
Parity						
	0	0 (0.00%)	0 (0.00%)	0 (0.00%)	11 (20.37%)	11 (12.22%)
	1	2 (18.18%)	5 (26.32%)	4 (66.67%)	23 (42.59%)	34 (37.78%)
	2+	9 (81.82%)	14 (73.68%)	2 (33.33%)	20 (37.04%)	45 (50.00%)
Menopause						
	Yes	10 (90.91%)	9 (47.37%)	4 (66.67%)	28 (51.85%)	51 (56.67%)
	No	1 (9.09%)	10 (52.63%)	2 (33.33%)	26 (48.15%)	39 (43.33%)
Metabolic syndrome						
	Yes	6 (54.55%)	12 (63.16%)	4 (66.67%)	35 (64.81%)	57 (63.33%)
	No	5 (45.45%)	7 (36.84%)	2 (33.33%)	19 (35.19%)	33 (36.67%)
Histological type						
	Endometrioid adenocarcinoma	11 (100.00%)	18 (94.74%)	5 (83.33%)	49 (90.74%)	83 (92.23%)
	Serous carcinoma	0 (0.00%)	0 (0.00%)	1 (16.67%)	3 (5.56%)	4 (4.44%)
	Clear cell carcinoma	0 (0.00%)	0 (0.00%)	0 (0.00%)	1 (1.85%)	1 (1.11%)
	Dedifferentiated cancer	0 (0.00%)	1 (5.26%)	0 (0.00%)	0 (0.00%)	1 (1.11%)
	Mesonephric-like adenocarcinomas	0 (0.00%)	0 (0.00%)	0 (0.00%)	1 (1.85%)	1 (1.11%)
FIGO stage						
	I∼II	9 (81.82%)	16 (84.21%)	4 (66.67%)	43 (79.63%)	72 (80.00%)
	III∼IV	2 (18.18%)	3 (15.79%)	2 (33.33%)	11 (20.37%)	18 (20.00%)
Myometrial invasion						
	<1/2	3 (42.86%)	11 (61.11%)	3 (50.00%)	22 (44.90%)	39 (48.75%)
	>1/2	2 (28.57%)	6 (33.33%)	3 (50.00%)	15 (30.61%)	26 (32.50%)
	No	2 (28.57%)	1 (5.56%)	0 (0.00%)	12 (24.49%)	15 (18.75%)
Vascular invasion						
	Yes	1 (10.00%)	2 (11.11%)	2 (33.33%)	6 (12.24%)	11 (13.25%)
	No	9 (90.00%)	16 (88.89%)	4 (66.67%)	43 (87.76%)	72 (86.75%)
Lymph node metastasis						
	Yes	0 (0.00%)	2 (11.11%)	1 (16.67%)	6 (13.64%)	9 (11.54%)
	No	10 (100.00%)	16 (88.89%)	5 (83.33%)	38 (86.36%)	69 (88.46%)

### Molecular characteristics of various molecular subtypes

#### *POLE* mut

*POLE* mutations were detected in 11 cases of EC, accounting for 12.2% of the sample. Notably, mutation hotspots were identified at P286R (5 cases), V411L (3 cases), A456P (2 cases), and S459F (1 case). Of these, one case exhibited concurrent mismatch repair deficiency (MMRd), presenting a dual molecular feature characteristic of LS, although LS screening did not reveal any germline MMR mutations. Furthermore, three cases were found to have clinically uncertain *TP53* class III variants. Additionally, two cases screened for LS displayed heterozygous variants of uncertain significance in the germline, specifically in *MLH1* and *MSH2* genes (Table 2).

**TABLE 2:** Molecular characteristics of the *POLE* mut subtypes.

No.	Molecular classification	Exon	Nucleotide substitution	Protein change	Abundance (%)	LS scring	*TP53* mut	dMMR
1	*POLE* mut	13	c.1231G > C	p.(V411L)	3.89	—	—	pMMR
2	*POLE* mut	14	c.1366G > C	p.(A456P)	2.52	—	—	pMMR
3	*POLE* mut	14	c.1366G > C	p.(A456P)	8.92	—	—	—
4	*POLE* mut	9	c.857C > G	p.(P286R)	37.82	—	—	pMMR
5	*POLE* mut	9	c.857C > G	p.(P286R)	11.96	*MLH1*, exon12 c.1153C > T p.(R385C), NM_000249.3, class 3	exon2 c.27C > T p.(S9=), 6.03%, class 3	—
6	*POLE* mut	9	c.857C > G	p.(P286R)	3.86	*MSH2*, exon14 c.2425G > A p.(E809K), NM_000251.3, class 3	—	pMMR
7	*POLE* mut	13	c.1231G > T	p.(V411L)	3.88	—	—	pMMR
8	*POLE* mut	9	c.857C > G	p.(P286R)	40.73	—	intron9 c.993 + 10C > A, 18.77%, class 3	pMMR
9	*POLE* mut	9	c.857C > G	p.(P286R)	11.96	—	—	—
10	*POLE* mut	14	c.1376C > T	p.(S459F)	20.97	—	exon8 c.817C > T p.(R273C), 13.46%, class 2	pMMR
11	*POLE* mut/MMRd	13	c.1231G > T	p.(V411L)	36.03	—	exon5 c.523C > T p.(R175C), 33.28%, class 3	pMMR

#### MMRd

This study identified 19 cases (21.1% of the sample) of the MMRd subtype in EC. Immunohistochemical analysis revealed a loss of MMR proteins in all these cases. Specifically, the loss was observed as follows: *MLH1* and *PMS2* concurrently in 9 cases (47.4%), *MSH2* and *MSH6* in 3 cases (15.8%), *MSH6* alone in 3 cases (15.8%), *PMS2* alone in 1 case (5.3%), and a combined loss of *MLH1*, *PMS2*, and *MSH2* in 1 case (5.3%). Additionally, 2 cases exhibited concurrent *TP53* gene mutations (dual molecular feature), with these mutations categorized as missense and frameshift mutations. 2 cases displayed clinically uncertain class III variants in the *POLE* gene, and 1 case showed a class III variant of uncertain clinical significance in the *TP53* gene. Significantly, 7 cases (36.8%) were diagnosed with LS. Notably, 2 of these cases had a family history of malignancy, both with maternal histories of colorectal cancer.

#### P53 abn

As shown in Table 3, p53 abn subtype was detected in 6 cases (6.7% of the sample). This included 4 cases with missense mutations, 1 case with a splice mutation, and 1 case with a nonsense mutation. IHC revealed p53 mutations in 3 of these cases. Furthermore, HER-2 IHC testing was conducted on 2 patients in FIGO stage III. The results showed 1 case with HER-2 (1+) and another with HER-2 (2+). Subsequent FISH (Fluorescence *In Situ* Hybridization) analysis on the patient with HER-2 (2+) did not reveal any HER-2 amplification. Screening for LS in all 6 patients with the p53 abnormality subtype showed no abnormalities. Among these patients, 5 underwent chemotherapy.

**TABLE 3:** Molecular characteristics of the p53 abn subtypes.

No.	Exon/intron	Nucleotide substitution	Protein change	Abundance	dMMR	p53 IHC
1	Exon7	c.743G > A	p.(R248Q)	missense	*PMS2-*	OE
2	Exon7	c.734G > T	p.(G245V)	7.92%	pMMR	Wild
3	Exon10	c.1024C > T	p.(R342*)	44.29%	pMMR	Wild
4	Exon6	c.577C > T	p.(H193Y)	59.57%	pMMR	CY
5	Exon8	c.817C > T	p.(R273C)	3.20%	pMMR	Wild
6	Intron6	c.672 + 1G > T	—	—	*MLH1*, *PMS2*	CA

* represents the stop codon.

#### NSMP

The NSMP subtype was identified in 54 cases, comprising 60% of 4 patient cohort. Within this subgroup, 2 cases were definitively diagnosed with LS, characterized by one *PMS2* germline mutation and one *MSH6* germline mutation, respectively. Additionally, 3 cases exhibited heterozygous germline VUS in MMR genes, including one *MLH1* germline variant, one *PMS2* germline variant, and one *MSH6* germline variant. Furthermore, one case was identified with a class III variant of uncertain clinical significance in the *TP53* gene.

### Results of LS screening

In our study involving 90 EC patients, pathogenic germline mutations in MMR genes were detected in 9 cases (10%). These mutations were distributed as follows: 3 cases with *MLH1* germline mutations, 2 cases with *PMS2*, 2 with *MSH2*, and 2 with *MSH6*. Among these 9 patients diagnosed with LS, molecular typing identified 7 cases as MMRd type and 2 as NSMP type. Abnormal MMR protein expression was observed in 7 of these cases. Notably, 2 patients with LS also reported a family history of malignancy after initial interviews (Table 4).

**TABLE 4:** Molecular and MMR protein IHC characteristics of LC-EC.

No.	Molecular classification	Mutated gene	Exon/intron	Nucleotide substitution	Protein change	dMMR	Other site
1	NSMP	*MSH6*	exon6	c.3523dup	p.(T1175Nfs*2)	*PMS2*	—
2	NSMP	*PMS2*	exon7	c.758_759del	p.(E253Gfs*12)	pMMR	—
3	MMRd	*MLH1*	exon2	c.132del	p.(T45Qfs*5)	—	—
4	MMRd	*PMS2*	exon10	c.1119_1122del	p.(Q374Sfs*10)	*MLH1*	—
5	MMRd	*MLH1*	intron13	c.1559-2A > T	—	*MLH1*	—
6	MMRd	*MSH6*	exon5	c.3261dup	p.(F1088Lfs*5)	*MSH6*	*POLE*, exon9 c.900C > T p.(I300=)
7	MMRd	*MSH2*	exon7	c.1276G > A	p.(G426R)	*PMS2*, *MSH2*	—
8	MMRd	*MLH1*	intron5	c.454-13A > G	—	*MLH1*, *PMS2*	—
9	MMRd	*MSH2*	exon4	c.680_681del	p.(R227Kfs*4)	*MSH2*, *MSH6*	—

* represents the stop codon.

Additionally, our study identified 6 cases with germline variants of uncertain clinical significance in MMR genes. These included 2 cases with *MLH1* variants, 1 with *MSH6*, 2 with *MSH6*, 1 with *PMS2*, and 1 with an *EPCAM* germline variant (Table 5).

**TABLE 5:** Molecular and MMR protein IHC characteristics of VUS.

No.	Molecular classification	Mutated gene	Exon/intron	Nucleotide substitution	Protein change	Other mutation	dMMR	p53 IHC
1	NSMP	*PMS2*	Intron12	c.2175-8T > C	p.(I637=)	—	pMMR	Wild
2	NSMP	*MLH1*	Exon17	c.1911T > C	p.(R385C)	—	pMMR	Wild
3	*POLE* mut	*MLH1*	Exon12	c.1153C > T	p.(R385C)	*POLE*, exon9 c.857C > G p.(P286R), class 2; *TP53*, exon2 c.27C > T p.(S9=), class 3	pMMR	Wild
4	*POLE* mut	*MSH2*	Exon14	c.2425G > A	p.(E809K)	*POLE*, exon9 c.857C > G p.(P286R), class 2	pMMR	Wild
5	NSMP	*MSH6*	Exon4	c.652A > G	p.(K218E)	—	—	CY
6	NSMP	*EPCAM*	Exon3	c.298G > A	p.(D100N)	—	—	Wild

## Discussion

Current methodologies for detecting *POLE* mut in EC encompass analysis of hotspot mutations and exonuclease domain pathogenicity. NGS is recommended for *POLE* mut detection when conditions permit. In our study, NGS was used to assess mutations in exons 9-14 of the *POLE* gene in 90 EC patients. This analysis identified 11 cases of *POLE* mutations, occurring in the common hotspots P286R (5 cases), V411L (3 cases), A456P (2 cases), and S459F (1 case). One case also exhibited concurrent MMRd with no germline PGVs in MMR genes. Two cases were identified with MMR heterozygous variants of uncertain significance through LS screening.

The relationship between *POLE* mut and MMRd is complex and not fully understood. Studies show that 3.4% of patients tested with TCGA molecular subtyping have concurrent *POLE* mut-MMRd. These patients’ prognosis is closer to those with only *POLE* mutations, suggesting they should be classified as *POLE* mut (León-Castillo et al., 2020). Additionally, approximately 35% of patients with *POLE* mutations also exhibit non-pathogenic *TP53* mutations (Vermij et al., 2022). In this study, three cases were found to have *TP53* class III variants of uncertain clinical significance, both missense and synonymous mutations. *TP53* gene mutations often occur secondary to *POLE* mut and MMRd, possibly due to impaired DNA polymerase proofreading and DNA damage repair, leading to decreased DNA replication fidelity and stability (Vermij et al., 2022).

When *POLE* gene testing indicates wild-type or non-pathogenic mutations, further testing for MMR proteins (IHC method) or MSI (PCR or NGS) is undertaken. In our study, 19 cases (21.1%) of MMRd type were detected, with two cases also exhibiting *TP53* mutation molecular features (dual molecular features). 8 cases (42.1%) were confirmed as LS. While PCR is the gold standard for MSI detection, it has limitations, including only 70% sensitivity for detecting *MSH6* germline mutations, leading to an increased application of NGS in MSI detection in recent years (Stelloo et al., 2017).

The status of p53 is generally determined by p53 protein expression (IHC method) or *TP53* gene mutation (NGS). The consistency between p53 protein IHC and *TP53* gene mutations is around 92.1% (Singh et al., 2020). Notably, subclonal expression, nonsense mutations, and low-proliferative tumor cells can result in focal weak positivity in P53 IHC, potentially misleading pathologists and leading to misinterpretation, as approximately 5% of p53 mutation subtype patients may not exhibit detectable P53 IHC mutations (Köbel et al., 2019). Inconsistencies between p53 IHC and NGS results are common in *POLE* mut and MMRd subtypes. Under these circumstances, P53 mutations do not predict poor prognosis (León-Castillo et al., 2020). Therefore, p53 IHC testing might misclassify about 15% of the copy-number high population into the NSMP group (Arciuolo et al., 2022). In our study, NGS was used to examine the entire coding region of the *TP53* gene, identifying 6 cases (6.7%) of the p53 abn subtype, with 3 cases detected by IHC. Relying solely on IHC can easily lead to misdiagnosis of the p53 abn subtype as NSMP.

Research suggests that high-grade endometrioid carcinoma patients with *TP53* gene mutations may benefit from HER-2 amplification and targeted therapy (Joehlin-Price et al., 2023). In our study, HER-2 status testing in two FIGO stage III p53 abn subtype patients did not reveal HER-2 amplification.

The NSMP subtype is the largest group in terms of proportion, characterized by significant heterogeneity in prognosis and molecular features. Current traditional morphological parameters and pathological assessments remain crucial for patient risk stratification (Momeni-Boroujeni et al., 2022). Molecular characteristics should be further refined for low-copy-number patients to provide more precise bases for prognosis and treatment. Studies have categorized NSMP patients into three groups based on different molecular features and their relationship with prognosis and clinicopathological characteristics: the first two groups are PTEN and PIK3CA mutation-type NSMP, and the third group is PTEN wild-type with mutations in either AKT1/KRAS/PIK3CA. The third group, mostly consisting of FIGO III/IV stage non-endometrioid cancers, has the worst prognosis (Momeni-Boroujeni et al., 2022). In our study, 54 cases (60%) of NSMP subtype were identified, with 2 cases diagnosed with LS, indicating that LS not only exists in MMRd but may also be present in other molecular subtypes.

Studies suggest that in EC-LS patients, *MSH2* mutations are the most common (50%–66%), followed by *MLH1* (24%–40%), *MSH6* (10%–13%), and *PMS2* (<5%) (Møller et al., 2017). Ren et al. (2020) identified 10 LS patients among 211 Chinese EC patients, with the most common mutation being *MSH6* (70%), followed by *MSH2* mutations (20%). In our study, 9 cases of LS were detected in 90 EC patients, including 3 *MLH1*, 2 *PMS2*, 2 *MSH2*, and 2 *MSH6* germline mutations. Among the 9 LS patients, 7 were classified as MMRd and 2 as NSMP. The routine screening method involves IHC for MMR proteins and further *MLH1* methylation testing for patients with *MLH1* loss, followed by germline mutation testing based on the results. However, in our study, 2 LS-EC patients were molecularly classified as NSMP, which could have been missed by standard screening methods. Additionally, 6 cases of MMR gene variants of uncertain clinical significance were identified in our study, currently lacking convincing evidence related to LS, representing a challenge and direction for exploration in LS screening.

Due to NGS testing providing more comprehensive molecular subtyping information and additional biological information beyond subtyping, the use of NGS to detect *POLE* gene mutations, MSI status, and *TP53* gene mutations for molecular subtyping has become the norm in China. However, the emergence of new pathogenic mutation sites and the heterogeneity of the NSMP subtype’s molecular characteristics pose new and higher demands on the design of NGS gene panels. Screening all newly diagnosed EC patients for MMR germline mutations can prevent misdiagnosis and missed diagnosis of LS-EC patients. However, due to the high cost of NGS testing and the small proportion of the target population, the clinical application of NGS testing for all EC-diagnosed patients requires further verification and exploration.

## Data Availability

The data presented in the study are deposited in the Dryad Digital Repository, the direct link is https://datadryad.org/stash/dashboard, and the accession number is 10.5061/dryad.83bk3jb24.
